# Can *Eretmocerus eremicus* Assess Oviposition Sites with Varying Host Densities and Predation Risks, and Make Decisions Based on Scent Cues?

**DOI:** 10.3390/insects17030329

**Published:** 2026-03-17

**Authors:** Luis Enrique Chavarín-Gómez, Víctor Parra-Tabla, Lizette Cicero, Carla Vanessa Sánchez-Hernández, Paola Andrea Palmeros-Suárez, Ricardo Ramírez-Romero

**Affiliations:** 1 Laboratorio de Control Biológico (LabCB-AIFEN), Departamento de Producción Agrícola, CUCBA, Universidad de Guadalajara, Zapopan 45100, Jalisco, Mexico; 2 Departamento de Ecología Tropical, Campus de Ciencias Biológicas y Agropecuarias, Universidad Autónoma de Yucatán, Apartado Postal 4-116, Itzimná, Mérida 97000, Yucatán, Mexico; 3 Laboratorio de Ecología Aplicada al Control Biológico (ECOBI), Instituto Nacional de Investigaciones Forestales, Agrícolas y Pecuarias (INIFAP), Mocochá 97454, Yucatán, Mexico; cicero.lizette@gmail.com

**Keywords:** wind tunnel, foraging behavior, host number, predation risk

## Abstract

Parasitoids use different signals to locate their hosts, and these signals can modulate their behavioral decisions. Thus, patch selection and foraging in patches with different characteristics depend on their ability to gather and use such information efficiently. In this study, we evaluated whether the parasitoid *Eretmocerus eremicus* (Hymenoptera: Aphelinidae), a natural enemy of *Trialeurodes vaporariorum* (Hemiptera: Aleyrodidae) on tomato plants (*Solanum lycopersicum*), uses scent cues to select and forage in patches that differ in host density and predation risk. Using choice bioassays in a wind tunnel under a continuous airflow, we recorded selection patch and selection time, as well as foraging parameters, including residence time, oviposition events, and attacks. Our results show that *E. eremicus* discriminated between sites with and without hosts using scent cues, but discrimination between patches with different host numbers was not detected under our assay conditions. It also distinguished between patches with maximum risk and those without risk, but not between subtle differences in risk. These findings suggest that *E. eremicus*, responded mainly to contrasting olfactory cues rather than to subtle odor differences. From an applied standpoint, our results motivate deeper investigation into how host- and predator-associated olfactory cues could fine-tune parasitoid deployment in biological control.

## Simple Summary

Parasitoid wasps can help control crop pests, but to do so effectively, they must detect where pests are and avoid places where predators could kill them. We tested whether the parasitoid wasp *Eretmocerus eremicus* relies on scent cues to select oviposition sites that optimize host encounters while minimizing predation risk. In laboratory choice tests conducted in a wind tunnel under a continuous airflow, we measured which patch the wasps selected, how long they took to decide, and their searching and oviposition behavior after arrival. The wasps clearly distinguished hosts from non-host patches using scent cues, but they did not reliably choose between patches with different host numbers under our assay conditions. They also avoided patches with very high predation risks more than they did safe patches, but they did not consistently respond to smaller differences in risk. Overall, under our experimental odor context, this parasitoid responded more clearly to strong scent contrasts than to subtle scent differences. Understanding how these wasps respond to host- and predator-related scent cues highlights the need for further research to identify which scent signals are most informative and how they could be used to improve parasitoid release planning for pest control.

## 1. Introduction

Biological control (BC) is a widely used strategy for managing agricultural pests, and among the most effective organisms employed are parasitoid insects [1,2]. These insects lay one or more eggs in, on, or near their host (the target pest), where they develop and ultimately cause host death [3]. During foraging, parasitoids generally encounter their hosts aggregated in patches, that is, sites with clustered hosts [4]. The number of ovipositions a parasitoid performs within these patches represents a significant component of its fitness [5], making the selection of an appropriate patch vital. In this context, parasitoids may rely on olfactory cues to locate, discriminate among, and choose between patches [6,7]. These cues may originate from plants, hosts, or some competitors and influence parasitoid decisions [8,9,10,11,12]. Studies have reported that the parasitoid *Eretmocerus eremicus* Rose and Zolnerowich (Hymenoptera: Aphelinidae), a natural enemy of several species of whiteflies, including *Trialeurodes vaporariorum* Westwood (Hemiptera: Aleyrodidae), can modify its foraging behavior depending on host number, patch-level predation risk, or the presence of competitors [13,14,15]. However, little is still known about how olfactory cues from patches that differ in host number and predation risk affect this parasitoid’s choices.

Parasitoids can rely on various environmental cues, such as volatile [6,7,11], acoustic [16], and visual [17] cues, to locate and discriminate among patches. These cues may allow them to identify and select higher-quality patches [18]. We can define patch quality based on the number of available hosts (the greater the number, the higher the quality) and the level of predation risk (the lower the risk, the higher the quality).

Thus, we can consider a patch with many hosts and a low predation risk to be high quality. This quality may influence parasitoid oviposition decisions [5,15,19,20]. For example, the parasitoid *E. eremicus* has been shown to modulate oviposition in response to host density and predation risk, with higher oviposition in patches with a higher host density and lower predation risk [15].

Odor cues, in turn, can be used by parasitoids to discriminate between patches with different host densities or to avoid competition with other parasitoids [8,9,10,12]. For example, it has been reported that the parasitoids *Cotesia glomerata* Linnaeus, 1758, and *Cotesia rubecula* Marshall, 1885 (Hymenoptera: Braconidae), were able to discriminate between patches with different host densities using odor cues originating from these patches [10]. Additionally, the response of the parasitoid *Stenomesius japonicus* Ashmead (Hymenoptera: Eulophidae) to odor cues emanating from the predator *Macrolophus pygmaeus* Rambur (Hemiptera: Miridae) has also been explored in olfactometry studies [12], which found that the parasitoid does not avoid risk using odor cues. However, another study that analyzed the response of the parasitoid *Trybliographa rapae* Westwood (Hymenoptera: Figitidae) to hosts infected with the fungi *Metarhizium brunneum* Petch and *Beauveria bassiana* (Balsamo) Vuillemin (Ascomycota: Hypocreales) found that these parasitoids avoided such hosts [21]. These results indicate that responses to odor cues can be variable.

Given that the ability to use odor cues can be key to improving reproductive performance, it is essential to evaluate this ability in species such as *E. eremicus*, a commercially available parasitoid used in biological control programs for whiteflies [22,23,24,25]. This species is widely used against *Bemisia tabaci* and has also been reported to be relevant to *T. vaporariorum* [22,23,24,25]. While foraging in patches, this parasitoid may encounter the generalist predator *Geocoris punctipes* Say (Hemiptera: Geocoridae), leading to an interaction known as intraguild predation (IGP) [13,14,26]. Previous work has shown that *E. eremicus* remains longer and oviposits more frequently in patches with higher host densities and lower predation risk [15]. However, it is unknown whether *E. eremicus* can use volatile cues to locate and select patches of varying quality (i.e., with more hosts and lower risk). If so, the knowledge generated could be used to optimize its performance, for example, by synthesizing compounds that act as attractants for highly infested sites [27] or by developing repellents for sites where IGP predators have been released. For this reason, the objective of the present study was to determine whether the parasitoid *E. eremicus* can use volatile cues from different patches to select for higher-quality patches, that is, those with a greater number of hosts and a lower predation risk.

In addition to its practical application in optimizing parasitoid efficacy, this research seeks to advance the knowledge of parasitoid ecology. Understanding how these insects integrate diverse environmental cues to make crucial decisions not only enables refinement of management strategies, but also deepens our knowledge of the mechanisms through which they interact with other organisms in multitrophic environments, thereby strengthening the conceptual basis of biological control from behavioral and ecological perspectives [28,29,30,31].

Based on the above, and considering that some parasitoids have exhibited a specific capacity to use volatile cues to discriminate between different host densities or to avoid competitors and infected hosts, we hypothesized that the parasitoid *E. eremicus* will use odor cues emanating from other patches to select those with a higher number of hosts or lower predation risk, that is, those of higher quality. Once a choice has been made, we predict that the parasitoid will remain longer, attack more, and oviposit more in patches with a higher host density or lower predation risk.

For this research, the biological model used included tomato plants (*Solanum lycopersicum*), a crop found in field or greenhouse conditions and affected by a variety of insect pests [32]. The pest insect used was *Trialeurodes vaporariorum*, the greenhouse whitefly, which is native to the Americas and is of worldwide importance [33,34,35]. As natural enemies, we used the parasitoid *Eretmocerus eremicus*, which is native to the Americas and has a preference for oviposition on second- and third-stage nymphs of whiteflies [36,37], and the predator *Geocoris punctipes*, whose distribution ranges from Canada to Panama [38]. This predator, in addition to attacking and consuming whitefly nymphs, can also consume immature and adult parasitoids, an interaction known as intraguild predation [13,14]. The predator and the parasitoid used in the present study are organisms produced and commercialized for the biological control of whiteflies [39,40].

## 2. Materials and Methods

The plants and insects used for colony maintenance and experiments were reared under controlled conditions of 24 ± 3 °C, 50 ± 10% relative humidity (RH), and a 14:10 h (light:dark) photoperiod [13,14].

### 2.1. Solanum lycopersicum

Tomato plants (*Solanum lycopersicum*) were obtained from commercial seeds purchased at La Casa del Hortelano (Guadalajara, Jalisco, Mexico). The seeds were sown directly in pots (9 cm height, 11 cm diameter) containing a 50:50 mixture of black soil and perlite, and the plants were irrigated with water and “Triple 18” fertilizer (0.8 g/L) every third day (SQM Comercial de México S.A. de C.V., Zapopan, Jalisco, Mexico). To prevent herbivore exposure, the plants were sheltered in spaces covered with an anti-aphid mesh. For herbivore colony maintenance (see below) and experiments, plants bearing 5–7 fully developed leaves were used [13,15].

### 2.2. Trialeurodes vaporariorum

Whitefly colonies maintained in the laboratory and used in experiments were established from virus-free individuals donated by Carla V. Sánchez-Hernández (Universidad de Guadalajara). Colonies were reared on tomato plants inside acrylic cages (38.5 × 30.0 × 45.0 cm), with all cage sides covered with an aphid-proof mesh. Taxonomic identification was performed by Vicente Carapia (Universidad Autónoma del Estado de Morelos). To obtain leaflets bearing second- and third-stage whitefly nymphs (hereafter referred to as “patches”), which are preferred by *E. eremicus* for oviposition [41,42], the method described by Chavarín-Gómez et al. [15] was followed. Tomato plants with 5–7 true leaves were placed inside acrylic cylinders, and 150 adult whiteflies were introduced and allowed to oviposit for 48 h. After this period, adult whiteflies were removed, and 14 days later, leaflets bearing second and third nymphs were obtained. From infested plants, leaflets (~3.5 cm long and ~2.5 cm wide) containing at least 35 whitefly nymphs were randomly taken. To keep the leaflets watered, 1 cm^3^ of Oasis^®^ floral foam (Nuevo Nova, Smithers-Oasis de México, S.A. de C.V., Nuevo Leon, Mexico) was attached to the petiole. To achieve the required number of whitefly nymphs per treatment, nymphs were carefully removed without damaging the leaflets using a DV4 stereomicroscope (Carl Zeiss, Oberkochen, Germany) and an entomological pin (#0.15, Entochrysis, Entomolab, CDMX, Mexico) until the desired density was reached.

### 2.3. Eretmocerus eremicus

The parasitoid *E. eremicus* was obtained from Koppert México S.A. de C.V. (Querétaro, Mexico) as parasitized nymphs of *T. vaporariorum* in cardboard. The cards containing parasitized nymphs were placed in acrylic cages (38.5 × 30.0 × 45.0 cm) to await adult emergence. The emerged adults were formed into cohorts and fed ad libitum with a solution of honey/water (7:3 mL) and 20 mL of water, which was applied to a paper towel (7 × 7 cm) inside a Petri dish. The adult stage of *E. eremicus* used in the experiments was 2–4 days old, given that they can mate, oviposit, and live for about 11 days from the first day of adulthood [43]. For the experiments, a parasitoid was picked up with a fine-bristled brush, placed in a glass tube, and verified as a female by observing the dimorphism in the shape of the parasitoid’s antennae with the aid of a stereoscope, and then transported to the release point of the flight tunnel.

### 2.4. Geocoris punctipes

*Geocoris punctipes* individuals were obtained from Koppert México S.A. de C.V. (Querétaro, Mexico) as fifth-instar nymphs. Upon arrival at our laboratory (LabCB-AIFEN), they were kept in polystyrene cages (40 cm × 30 cm × 31 cm) and fed ad libitum with pollen (5 g), sorghum (10 g) (Apiarios Rancaño, Mexico City, Mexico, and UDG-110, Zapopan, Jalisco, Mexico), water (20 mL applied to a 7 × 7 cm paper towel), and 5 g of artificial diet [44]. The artificial diet and water were changed daily, while the pollen and sorghum were changed once a week [13,15]. Females and males were kept together in the polystyrene cages until use. The adult females used in the experiments were between 8 and 20 days old, taking into account their premating and preoviposition periods, as well as their life expectancy (~108 days as adults) [45,46].

### 2.5. Experimental Setup

A flight tunnel was constructed for the experiments (Supplementary Figure S1A,B), which was based on previous olfactometry and flight tunnel studies [11,47,48,49]. The purpose of this device was to observe the selection patch and collect information on foraging decisions after the selection had been made. The flight tunnel was made of acrylic with the following dimensions: 25 cm long × 10 cm wide × 3.5 cm high, with a 12.5 cm longitudinal division at the rear of the tunnel (creating two spaces called “Field 1” and “Field 2”) (Supplementary Figure S1A). A fan and carbon filter (Campanas Fersai, Mexico City, Mexico) were installed at the rear of the tunnel to filter air and remove potential odors before they enter the tunnel (Supplementary Figure S1B). Before formal bioassays, airflow behavior in the flight tunnel was verified using dry ice as a visible tracer. These checks showed that airflow streams from the two odor fields remained separated and were continuously displaced downstream, with no evident retention within the observation area. The experiments were conducted under controlled conditions at 24 ± 3 °C and 50 ± 10% relative humidity (RH). Based on a study with *Encarsia formosa* [50] and preliminary tests, it was determined that the wind speed should be set to 0.4 m/s using an anemometer (Krestrel 2000, Kestrel, Boothwyn, PA, USA). This speed maintained a continuous airflow without affecting the parasitoid’s movement in the tunnel. The front part of the flight tunnel was covered with organdy [51]. On the upper side, 1 cm from the start of the tunnel, there was a 1 cm diameter hole covered with parafilm (PARAFILM^®^, ProLab S.A. de C.V., Tlajomulco de Zuñiga, Jalisco, Mexico) through which the parasitoid was introduced. Two LED lamps were used to illuminate the tunnel, placed perpendicular to the flight tunnel, and provided 4500 lux at the tunnel’s center. Foraging parameters were observed through the flat acrylic top of the flight tunnel with a Stemi 2000-C stereoscope (Carl Zeiss, Oberkochen, Germany) connected to an Axio Vision 4.8 camera (Carl Zeiss, Oberkochen, Germany), and behaviors were recorded using Etholog 2.2 software [52].

### 2.6. Experiment 1: Effect of the Number of Hosts

The objective of this experiment was to determine whether the parasitoid can use volatile signals emitted by patches with different numbers of hosts to select the patch with the highest number of hosts. It also sought to determine its foraging behavior (i.e., residence time, number of oviposition events, number of attacks, oviposition time, and attack time) in the patch once its choice had been made. A completely randomized design was followed with the number of hosts as the factor of interest (factor levels: 0, 2, and 32 hosts). The treatments prepared were: (1) the control patch consisting of a leaflet without nymphs (hereinafter referred to as “control”), (2) the low number patch consisting of a leaflet with 2 nymphs (hereinafter referred to as “low number”), and (3) the high number patch consisting of a leaflet with 32 nymphs (hereinafter referred to as “high number”). Once the treatments were prepared, they were placed in pairs in the flight tunnel to determine the parasitoid’s ability to discriminate between patches. The pairs tested were: control vs. low number; control vs. high number; and low number vs. high number.

For this experiment, the patches corresponding to each treatment were prepared 24 h before the observations began [15]. Before starting the observation, the treatments corresponding to each paired combination were placed inside the tunnel, and the field (see Supplementary Figure S1A) in which the treatments were assigned was chosen at random. Observations were made between 09h00 and 18h00. Once the flight tunnel was prepared, a female parasitoid was released, and the observation began. If the parasitoid did not choose and/or did not enter any patch within 70 min (the average time it took for the parasitoid to make a choice in a pilot experiment), it was discarded, and a new replicate was prepared (new flight tunnel and new organisms, leaflets with nymphs and parasitoids).

The response variables recorded were: (1) selection patch, defined as the first patch entered by the parasitoid after the onset of observation in each replicate; (2) selection time, measured as the time (in seconds) between the introduction of the parasitoid into the wind tunnel and its entry into a patch; (3) residence time, defined as the time (in seconds) the wasp remains within a patch before leaving, excluding brief exits lasting less than 60 s [15,53]; (4) number of attacks, where each attack was characterized by the insertion of the ovipositor under the host and lasted less than 50 s [15,37]; (5) number of oviposition events, defined as ovipositor insertions that exceeded 50 s in duration [15,37]; and (6) attack and oviposition durations, measured as the total time spent in each respective behavioral event. The observation ended when the parasitoid left the patch for more than 60 s. [15,53]. If the parasitoid did not choose and/or did not enter any patch within 70 min (the average time it took for the parasitoid to make a choice in a pilot experiment), it was discarded, and a new replicate was prepared (new flight tunnel and new organisms, leaflets with nymphs and parasitoids). A different parasitoid was used for each combination tested, and each combination was replicated 29 times.

#### Data Analysis of Experiment 1

The analysis of each response variable was conducted separately for each paired comparison (i.e., control vs. low number, control vs. high number, and low number vs. high number), including verification of the assumptions of normality and homoscedasticity. Data that met these assumptions were analyzed using *t*-tests [54]. When the assumptions were not met for a given response variable, the variable was either transformed (details below) or analyzed using a generalized linear model (GLM) (MASS package) [55]. The analysis of the variable “selection patch” was performed for each paired comparison (i.e., control vs. low number, control vs. high number, and low number vs. high number) using a two-tailed test of equality of proportions under the null hypothesis that the proportion of patches chosen was equal between the offered patches. The variables “selection time” and “residence time” were log transformed (x + 1) and analyzed using *t*-tests for the three paired comparisons. The variables “number of oviposition events” and “number of attacks” were analyzed using GLMs with a negative binomial distribution for the low number vs. high number comparison (MASS package) [55]. These variables were not analyzed for the control vs. low-number and control vs. high-number comparisons because nymphs were absent in the controls. Similarly, “oviposition duration” was analyzed using a GLM with a gamma distribution, and “attack duration” was analyzed using a *t*-test on the sqrt (x + 0.5) transformed variable for the low number vs. high number comparison; these variables were not analyzed for the other two comparisons because of the absence of nymphs in the controls. All analyses were performed in R v.3.5.1 [56].

### 2.7. Experiment 2: Effect of the Predation Risk

The objective of this bioassay was to determine whether the parasitoid could use the aromatic signals emitted by patches with different levels of predation risk, select the highest-quality patch, and characterize its foraging behavior once the patch had been chosen. A completely randomized design was established, with predation risk as the factor of interest (three levels: control, low risk, and maximum risk). The level of risk was associated with the number of predator-related signals (e.g., excreta or eggs) on the leaflet left by predator female *G. punctipes* over a 24 h period inside the glass vial before the observations; the more signals a patch contained, the higher its predation risk, as reported by Chavarín-Gómez et al. [15]. For this experiment, all patches contained 8 second- and third-stage whitefly nymphs. The treatments tested were: (1) the control patch, which consisted of a leaflet with eight whitefly nymphs without predator-related signals (hereinafter referred to as “control”); (2) the patch with a low risk of predation, consisting of a leaflet with 8 whitefly nymphs + predator cues [e.g., excreta or eggs of the predator] (hereinafter referred to as “low risk”); and (3) the patch with the maximum risk of predation, consisting of a leaflet with 8 whitefly nymphs + predator cues [e.g., excreta or eggs of the predator] + *G. punctipes* present + four male parasitoids that had been previously predated (hereinafter referred to as “maximum risk”) [15]. For treatment preparation, leaflets containing ~35 whitefly nymphs were placed in glass vials (4.5 cm high × 2.5 cm in diameter), a female *G. punctipes* was added, and the vials were left for 24 h before observations. The same procedure was followed in the control patch, but the IG predator was not added [13,15]. Minutes before the observation began, the leaflets were removed from the glass vials, and the number of whitefly nymphs was adjusted to eight under a stereoscope, following the procedure described in Section 2.2. The patches were then placed randomly in the flight tunnel in the following combinations: (i) control vs. low risk, (ii) control vs. maximum risk, and (iii) low risk vs. maximum risk. Once the corresponding patches were placed in the tunnel, the air speed was stabilized, and a female parasitoid was introduced as described in Section 2.6. The response variables recorded (as defined in Section 2.6) were: (1) selection patch, (2) selection time, (3) residence time, (4) number of oviposition events, (5) oviposition duration, (6) number of attacks, and (7) attack duration. The observation ended when the parasitoid left the patch for more than 60 s [15,53]. If the parasitoid did not choose and/or did not enter any patch within 70 min, it was discarded, and a new replicate was prepared (new flight tunnel and new organisms). For each replicate, a clean wind tunnel and new organisms were used, and each combination was replicated 29 times.

#### Data Analysis of Experiment 2

The assumptions of normality and homoscedasticity were verified for each paired combination [54] following the procedure described in Section Data Analysis of Experiment 1. The variable “selection patch” was analyzed with a two-tailed test of equality of proportions, with the null hypothesis being that both patches were chosen in equal proportions by the parasitoid in each paired combination (i.e., control vs. low risk, control vs. maximum risk, and low risk vs. maximum risk). The variables “selection time” and “residence time” were analyzed using *t*-tests across the three paired comparisons. The “number of oviposition events” was analyzed using a *t*-test after log(x + 1) transformation for the control vs. low-risk comparison and without transformation for the other two comparisons. Finally, the “number of attacks,” “oviposition duration,” and “attack duration” were analyzed using *t*-tests across the three paired comparisons. All analyses were performed in R v. 3.5.1 [56].

## 3. Results

### 3.1. Effect of the Number of Hosts

**Figure 1:**
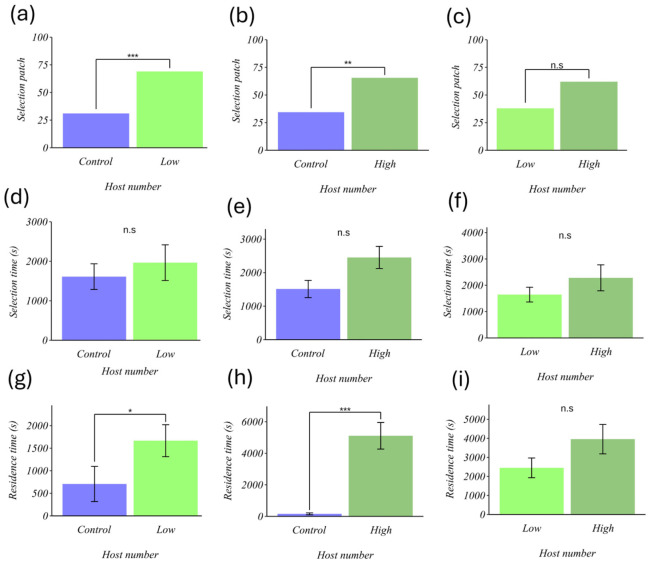
Effect of host number on the foraging behavior of *Eretmocerus eremicus*. (**a**) selection patch (control vs. low number), (**b**) selection patch (control vs. high number), (**c**) selection patch (low number vs. high number), (**d**) selection time (control vs. low number), (**e**) selection time (control vs. high number), (**f**) selection time (low number vs. high number), (**g**) residence time (control vs. low number), (**h**) residence time (control vs. high number), (**i**) residence time (low number vs. high number). (**d**–**i**) Each column represents the mean (±SE). The horizontal axis shows the levels of the host number factor (control = 0 nymphs, low = 2 nymphs, and high = 32 nymphs). * *p* < 0.05, ** *p* < 0.01, *** *p* < 0.001, n.s.= not significant.

Our results show that the parasitoid chose patches with hosts more frequently than patches without hosts (control vs. low number: χ^2^ = 6.89, df = 1, *p* = 0.008; control vs. high number: χ^2^ = 9.68, df = 1, *p* = 0.03; Figure 1a,b). However, it was observed that it selected patches indiscriminately, regardless of host density (low vs. high: χ^2^ = 2.48, df = 1, *p* = 0.11; Figure 1c). Regarding the time it took to choose, we found that it was similar across the different treatments and combinations (control vs. low number: t = 0.63, df = 9.16, *p* = 0.54; control vs. high number: t = 1.07, df = 22.71, *p* = 0.29; low number vs. high number: t = 0.15, df = 26.15, *p* = 0.87; Figure 1d,e, and f, respectively). These results indicate that when the parasitoid faces volatiles from patches with and without hosts, it significantly prefers those with hosts; however, when both patches have hosts, no significant preference was detected between low- and high-density patches, and takes a similar amount of time to make a choice under our assay conditions.

Regarding the time spent in the patch, we found that it was significantly longer in patches with hosts compared to the control without hosts (control vs. low number: t = 2.54, df = 12.18, *p* = 0.02; control vs. high number: t = 6.87, df = 19.51, *p* < 0.001, Figure 1g,h). However, when comparing patches with low and high host numbers, the residence time did not differ significantly (low vs. high: t = 0.25, df = 24.65, *p* = 0.79; Figure 1i). These results indicate that the parasitoid spends substantially more time in patches with hosts than in those without. Still, the time spent in patches with few and many hosts did not differ significantly.

**Figure 2:**
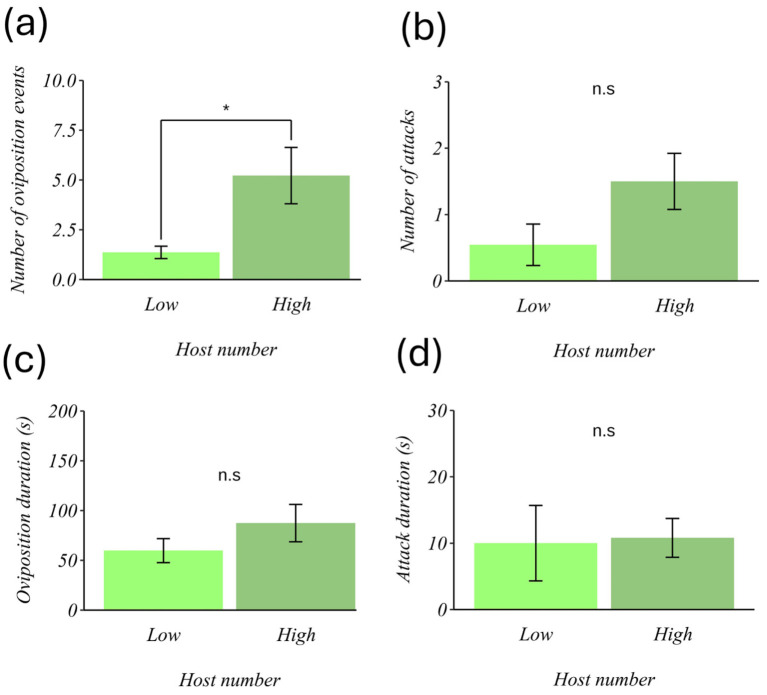
Effect of host number on the foraging behavior of *Eretmocerus eremicus*. Each column represents the mean (±SE). (**a**) Number of oviposition events, (**b**) oviposition duration (in seconds), (**c**) number of attacks, and (**d**) attack duration (in seconds). The horizontal axis shows the levels of the host number factor (low = 2 nymphs and high = 32 nymphs). * *p* < 0.01, n.s. = not significant.

Concerning the number of oviposition events, we found that the parasitoid oviposited more in patches with a high host density than in those with a low host density (χ^2^ = 5.96, gl = 1, *p* = 0.014; Figure 2a). However, the number of attacks, the duration of oviposition, and the duration of the attack did not show significant differences (χ^2^ = 2.44, df = 1, *p* = 0.11; χ^2^ = 0.95, df = 1, *p* = 0.24; t = 0.570, df = 18.20, *p* = 0.575; Figure 2b–d, respectively).

### 3.2. Effect of the Predation Risk

**Figure 3:**
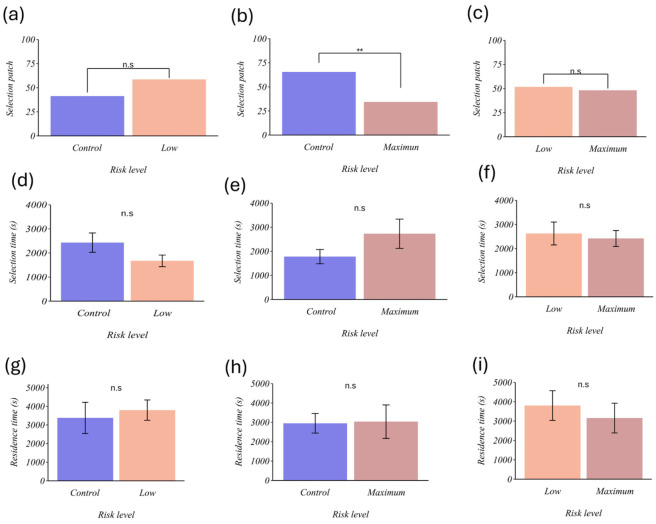
Effect of predation risk on the foraging behavior of *E. eremicus*. (**a**) selection patch (control vs. low risk), (**b**) selection patch (control vs. maximum risk), (**c**) selection patch (low risk vs. maximum risk), (**d**) selection time (control vs. low risk), (**e**) selection time (control vs. maximum risk), (**f**) selection time (low risk vs. maximum risk), (**g**) residence time (control vs. low risk), (**h**) residence time (control vs. maximum risk), (**i**) residence time (low risk vs. maximum risk). (**d**–**i**) Each column represents the mean (±SE). The horizontal axis shows the levels of the predation risk factor (control = 8 nymphs, low risk = 8 nymphs + IG predator excreta, and maximum risk = 8 nymphs + IG predator excreta + presence of IG predator + conspecifics of the parasitoid). ** *p* < 0.001, n.s.= not significant.

Our results show that parasitoids significantly preferred patches without risk signals (control) over patches with maximum risk signals (χ^2^ =4.41, gl = 1, *p* = 0.03, Figure 3b). However, patch choice did not differ when one of the options contained few risk signals (control vs. low risk: χ^2^ = 1.10, df = 1, *p* = 0.29; low risk vs. maximum risk: χ^2^ = 1, df = 1, *p* = 1, Figure 3a,c). In terms of selection time, parasitoids took similar times to choose (control vs. low risk: t = 1.61, df = 18.67, *p* = 0.12; control vs. maximum risk: t = 1.40, df = 13.42, *p* = 0.18; low risk vs. maximum risk: t = 0.35, df = 24.57, *p* = 0.72; Figure 3d,e, and f, respectively). Similarly, parasitoids spent similar amounts of time in the different treatments (control vs. low risk: t = 0.41, df = 19.83, *p* = 0.68; control vs. maximum risk: t = 0.08, df = 15.29, *p* = 0.93; low risk vs. maximum risk: t = 0.59, df = 26.96, *p* = 0.55; Figure 3g–i, respectively). Regarding the response variables, number of oviposition events, oviposition duration, number of attacks, and attack duration, no significant differences were found when comparing the treatments in the different combinations (Table 1).

## 4. Discussion

The objective of this study was to determine whether the parasitoid *E. eremicus* could use aromatic signals and select patches based on host number and predation risk. Considering that some parasitoids use volatile signals to modify their behavior and choose different host densities or avoid competitors, we hypothesized that *E. eremicus* would use aromatic signals to select the highest quality patches (i.e., those with the highest number of hosts and lowest risk of predation). Our results supported the hypothesis under specific scenarios. For example, when the parasitoid was exposed to signals from patches with or without hosts, it significantly preferred those with hosts. When exposed to patches with vs. without predator-associated signals, it chose the patches without signals. However, when the comparison was between patches with different numbers of hosts or predator-associated signal strengths, the parasitoid selected patches indiscriminately. These results suggest that this parasitoid is capable of discriminating and choosing based on the presence/absence of aromatic signals but not based on different strengths of these signals.

### 4.1. Effect of the Number of Hosts

Previous studies have shown that some parasitoids, such as *Cotesia glomerata*, *C. rubecula* (Hymenoptera: Braconidae), and *Ibalia leucospoides* (Hymenoptera: Ibaliidae), can discriminate between patches of different quality (determined by the number of hosts) using volatile signals [10,18]. Therefore, in this study, we propose the hypothesis that *E. eremicus* can use volatile signals to select patches with the highest host densities. Our results showed that the parasitoid *E. eremicus* used aromatic signals and significantly preferred patches with hosts over those without. This result indicates that *E. eremicus* discriminates based on the presence of hosts using aromas, probably terpenes, sesquiterpenes, alcohols, monoterpenes, or esters, among others, that are released when the whitefly feeds on the tomato plant [57]. In contrast, when patches with different host densities (low vs. high) were presented, no significant differences in choice were detected under our assay conditions. At present, this result is best interpreted as no detectable discrimination between host density treatments in this assay; whether odor dynamics after host density adjustment contributed to this outcome remains a plausible but untested hypothesis (discussed below). Thus, *E. eremicus* responded to the aromas associated with host presence versus absence, whereas discrimination between low and high host density was not expressed in this assay. This pattern may indicate that the parasitoid responds to relatively low thresholds for aromatic signals of host presence, but not necessarily to finer quantitative differences in cue intensity under the tested conditions. This pattern contrasts with that reported by Geervliet et al. [10] and Fischbein et al. [18], who noted that parasitoids of the genera *Cotesia* (Hymenoptera: Braconidae) and *Ibalia* (Hymenoptera: Ibaliidae) discriminated against patches of higher quality (i.e., with a greater number of hosts). These contrasting results may be related to parasitoid perception thresholds and/or to differences in the nature, composition, and temporal dynamics of emitted odors. Regarding odor dynamics specifically, one possibility is that low- and high-density patches retained a similar induced-plant volatile background after host density adjustment, which could have reduced the olfactory contrast between treatments. However, available evidence also indicates that post-herbivory volatile emissions can be dynamic, with declines in several compounds over short post-removal intervals (e.g., 6–24 h) [58,59,60,61,62]. In addition, because the assay was conducted in a wind tunnel with a continuous airflow, odor plumes were continuously renewed rather than allowed to accumulate in a closed arena. Therefore, although persistent olfactory equivalence between patch types for 1–2 days is plausible, this hypothesis should be tested directly by characterizing VOCs across treatments and over time after density adjustment. However, it is also necessary to consider that our results are consistent with those of Liu et al. [63], who found that *Venturia canescens* (Hymenoptera: Ichneumonidae) showed no preference between patches with different host densities or related odor signals. These results suggest that some parasitoids may use odor cues to discriminate between patches based on host density, whereas other species, as in our case, only do so under specific conditions of signal presence or absence [63]. Thus, it is possible that, when released in greenhouses or in the field, *E. eremicus* could use the presence/absence of volatiles to approach host patches, thereby optimizing host location. However, it is essential to consider that, under field conditions, volatile signals may disperse, degrade, or interact with other stimuli [64,65]. Hence, these observations should be evaluated under greenhouse or field conditions to better determine the role and potential applications of aromatic signals in optimizing the use of *E. eremicus*.

When analyzing the time *E. eremicus* took to select a patch, we expected it to be shorter for patches with a higher host number. However, we observed that the time required was approximately the same across host counts. At least two possible explanations could account for this observation. On the one hand, this parasitoid may spend a relatively constant amount of time processing and recognizing signals before making a decision, regardless of the concentration, intensity, or type of aromatic signal. Alternatively, the aromatic signals between treatments may have been similar in concentration or type, so they did not produce detectable differences in decision times. Evaluating choice times across different concentrations or types of aromas would help determine whether either possibility is at work.

Once the choice had been made, our prediction was that the parasitoid would remain longer, attack more, and perform more ovipositions in patches with a greater number of hosts [15]. Indeed, we found that *E. eremicus* remained longer in patches with hosts than in those without hosts and performed more oviposition events in patches with more hosts. However, the oviposition duration, the number of attacks, and the attack duration did not differ significantly between patches with few and many hosts (2 vs. 32). Additionally, when faced with patches with different numbers of hosts (2 vs. 32), residence times were also similar, which contrasts with the results of Chavarín-Gómez et al. [15], which were obtained in a no-choice context. These results indicate that, in a choiceless context, the parasitoid focuses on exploiting the only available patch, adjusting its residence time and number of attacks to the number of hosts present [15]. In contrast, in a choice context, where the parasitoid is simultaneously exposed to two patches with different numbers of hosts (2 vs. 32), once the existence of both patches is detected, *E. eremicus* may tend to distribute its residence times more evenly between them to exploit both available patches. Testing this hypothesis, for example, by evaluating behavior in patches with different host densities located in the same patch or plant, would help clarify the decision-making mechanisms underlying their foraging strategy [66,67].

In the context of biological control, the results obtained suggest that when searching for oviposition sites, *E. eremicus* would preferentially choose those where it perceives signals associated with the presence of whitefly nymphs. This behavior could represent an advantage in foraging efficiency. However, the fact that the parasitoid spends similar amounts of time in patches with 2 or 32 hosts suggests a potential disadvantage. Devoting similar amounts of time to searching in patches with low and high host numbers could reduce its efficiency and, consequently, affect its performance as a control agent. The balance between efficient selection of patches at a distance (based on the detection of volatiles) and prolonged permanence in patches with few hosts is a topic to be explored. However, the information the parasitoid obtains at a distance could play a key role in its foraging strategy, directly affecting its reproductive success and its ability to control whiteflies.

### 4.2. Effect of the Predation Risk

Based on previous studies evaluating whether parasitoids use volatile signals associated with predation or infection risk [12,21], we hypothesized that *E. eremicus* would be able to discriminate between patches with varying predation risk using these signals. Our results show that, when faced with the control vs. maximum-risk choice, the parasitoid significantly selected the risk-free patches more often (Figure 3b). In contrast, when faced with control vs. low risk or low risk vs. maximum risk, the choice was similar across patches. Taken together, these results indicate that, in our essays, risk avoidance was detected primarily under highly contrasting risk-cue contexts (control vs. maximum risk), but not when the options differed more subtly in risk cues (control vs. low risk).

This observed behavior of *E. eremicus* may indicate that its discrimination depends on perceived contrast rather than risk level. In other words, the selection of the control patch over the maximum-risk patch suggests that the presence of different cues (i.e., predator excreta, the predator itself, and preyed upon parasitoids) constitutes a sufficiently strong dissuasive stimulus under our assay conditions. In contrast, when comparing control vs. low risk, no preference was detected, possibly because predator excreta alone (low risk) may represent a weaker or more ambiguous signal [68,69], perhaps confounded with plant- or host-derived cues, or dissipating rapidly [65,70]. Alternatively, one of the three signals presented in the maximum-risk condition may have produced the dissuasive stimulus. Subsequent studies analyzing the effects that these signals, individually or in combination, have on the parasitoid’s behavior would allow us to determine whether the observed response is triggered by a particular signal or by a combination of signals. Finally, when faced with the dichotomy of “low risk vs. maximum risk,” the parasitoid showed no preference, suggesting risk acceptance. Upon perceiving risk in both patches, it chose indiscriminately.

Once the parasitoid entered a patch, it was expected to remain and oviposit less in the highest-risk patches, as was observed in experiments without a choice [15]. However, in the present study, the parasitoid showed similar residence times and numbers of oviposition events across the three treatments. As mentioned previously, under choice conditions, information from the other patch can modulate the parasitoid’s behavior, leading it to distribute its foraging time more evenly.

Ecologically, the results suggest that the parasitoid can detect risk signals (e.g., predation on congeners) and use them to determine whether to enter a patch under strongly contrasting cue contexts. However, once it chooses between the two patches (both containing eight hosts), it appears to prioritize oviposition over risk avoidance as it spent similar amounts of time in the low- and maximum-risk patches. At first glance, this strategy does not seem advantageous. However, it would be necessary to evaluate whether this behavior provides any other adaptive benefit against IG predators. To explore this, whitefly nymph densities could be assessed alongside the IG predator and the parasitoid, and offspring produced under varying levels of risk and host availability could be quantified. Finally, in the context of biological control, the results indicate that *E. eremicus* avoids sites with the highest predation risk, suggesting that, in the field, it would tend to colonize sites with no or low predation risk first. However, when choosing between low- and maximum-risk sites, the parasitoid would not be expected to show a consistent preference based on our assays, prioritizing oviposition over risk.

## 5. Conclusions

Our results showed that *E. eremicus* can discriminate between patches with and without hosts using volatile signals, whereas discrimination between low- and high-host-density patches was not detected under our assay conditions. The timing of the selection was similar across treatments. The residence time was only longer when the alternative lacked hosts, while it was comparable between patches with 2 and 32 hosts. Even so, the number of oviposition events was higher in patches with 32 hosts. Regarding predation risk, the parasitoid avoided the highest-risk patches and only employed volatile signals when a risk-free alternative was available, showing no clear preference between low- and high-risk patches. Overall, the results indicate that, in our experimental system, *E. eremicus* relies primarily on clear contrasts in odor cues, such as the presence or absence of hosts or the highest risk, rather than on subtle scent gradients, and that, once within a patch, it prioritizes host exploitation over assessing predation risk.

## Figures and Tables

**Table 1:** Results of the *t*-test for each combination for the variables number of oviposition events, oviposition duration, number of attacks, and attack duration in the predation risk bioassay (α = 0.05). control = 8 nymphs, low risk = 8 nymphs + predator cues, and maximum risk = 8 nymphs + predator cues + predator presence + conspecifics of the parasitoid preyed upon by the predator.

Variable	Combination	Statistics
Number of oviposition events	control vs. low risk	t = −0.375, df = 22.869, *p* = 0.710
	control vs. maximum risk	t = 0.112, df = 17.657, *p* = 0.911
	low risk vs. maximum risk	t = −0.387, df = 26.659, *p* = 0.701
Oviposition duration	control vs. low risk	t = −0.611, df = 21.316, *p* = 0.547
	control vs. maximum risk	t = 0.297, df = 23.272, *p* = 0.768
	low risk vs. maximum risk	t = −0.527, df = 26.196, *p* = 0.602
Number of attacks	control vs. low risk	t = −0.564, df = 26.845, *p* = 0.577
	control vs. maximum risk	t = −0.164, df = 22.517, *p* = 0.870
	low risk vs. maximum risk	t = −0.971, df = 19.638, *p* = 0.343
Attack duration	control vs. low risk	t = −0.623, df = 25.059, *p* = 0.538
	control vs. maximum risk	t = −0.328, df = 20.88, *p* = 0.746
	low risk vs. maximum risk	t = −1.034, df = 26.612, *p* = 0.310

## Data Availability

The raw data supporting the conclusions of this article will be made available by the authors on request.

## Supplementary Materials

The following supporting information can be downloaded at: https://www.mdpi.com/article/10.3390/insects17030329/s1, Supplementary Figure S1A: Flight tunnel with sections depicted, including the release point, the acrylic division, field 1, and field 2. Supplementary Figure S1B: Flight tunnel placed over the stereoscope with the lighting system. The fan location is visible, as is the area where the parasitoid can explore the scents associated with the offered patches.

## References

[1] Abeijon L.M., Gómez-Llano J.H., Ovruski S.M., Garcia F.R. (2026). Global Distribution of Three Parasitoids of *Drosophila suzukii* (Diptera, Drosophilidae): Present and Future Climate Change Scenarios. Insects.

[2] Kalyanasundaram M., Kamala I.M., Omkar (2016). Parasitoids. Ecofriendly Pest Management for Food Security.

[3] Hoy M.A., Metcalf R., Luckmann W.H. (1994). Parasitoids and predators in management of arthropod pests. Introduction to Insect Pest Management.

[4] Heimpel G.E., Rosenheim J.A., Mangel M. (1996). Egg limitation, host quality and dynamic behavior by parasitoid in the field. Ecology.

[5] Goubault M., Fourrier J., Krespi L., Poinsot D., Cortesero A.M. (2004). Selection strategies of parasitized hosts in a generalist parasitoid depend on patch quality but also on host size. J. Insect Behav..

[6] Lozano C., González E., Peña A., Campos M., Plaza M.T., Rodríguez M., Izquierdo I., Tamayo J. (2000). Response of parasitoids *Dendrosoter protuberans* and *Cheiropachis quadrum* to attractants of *Phloeotribus scarabeoides* in an olfactometer. J. Chem. Ecol..

[7] Ayelo P.A., Yusuf A.A., Chailleux A., Mohamed S.A., Pirk C.H.W., Deletre E. (2022). Chemical cues from honeydew and cuticular extracts of *Trialeurodes vaporariorum* serve as kairomones for the parasitoid *Encarsia formosa*. J. Chem. Ecol..

[8] Castelo M.K., Corley J.C., Desouhant E. (2003). Conspecific avoidance during foraging in *Venturia canescens* (Hymenoptera: Ichneumonidae): The roles of host presence and conspecific densities. J. Insect Behav..

[9] Janssen A., Van Alphen J.J.M., Sabelis M.W., Bakker K. (1995). Specificity of odour-mediated avoidance of competition in *Drosophila* parasitoids. Behav. Ecol. Sociobiol..

[10] Geervliet J.B.F., Ariens S., Dicke M., Vet L.E.M. (1998). Long-distance assessment of patch profitability through volatile infochemicals by the parasitoids *Cotesia glomerata* and *C. rubecula* (Hymenoptera: Braconidae). Biol. Control.

[11] Ramírez-Romero R., Sivinski J., Copeland C.S., Aluja M. (2012). Are individuals from thelytokous and arrhenotokous populations equally adept as biocontrol agents? Orientation and host searching behavior of a fruit fly parasitoid. Biol. Control.

[12] Chailleux A., Wajnberg E., Zhou Y., Amiens-Desneux E., Desneux N. (2014). New parasitoid-predator associations: Female parasitoids do not avoid competition with generalist predators when sharing invasive prey. Naturwissenschaften.

[13] Velasco-Hernández M., Ramirez-Romero R., Cicero L., Michel-Rios C., Desneux N. (2013). Intraguild predation on the whitefly parasitoid *Eretmocerus eremicus* by the generalist predator *Geocoris punctipes*: A behavioral approach. PLoS ONE.

[14] Bao-Fundora L., Ramirez-Romero R., Sánchez-Hernández C.V., Sánchez-Martínez J., Desneux N. (2016). Intraguild predation of *Geocoris punctipes* on *Eretmocerus eremicus* and its influence on the control of the whitefly *Trialeurodes vaporariorum*. Pest. Manag. Sci..

[15] Chavarín-Gómez L.E., Torres-Enciso P., Palmeros-Suarez P.A., Ramírez-Romero R. (2023). Influence of the number of hosts and the risk of predation on the foraging behavior of the parasitoid *Eretmocerus eremicus*. Pest Manag. Sci..

[16] Sakaguchi K.M., Gray D.A. (2011). Host song selection by an acoustically orienting parasitoid fly exploiting a multispecies assemblage of cricket host. Anim. Behav..

[17] Fisher S., Samitz J., Wäckers F.L., Dorn S. (2001). Interaction of vibrational and visual cues in parasitoid host location. J. Comp. Physiol..

[18] Fischbein D., Bettinelli J., Bernstein C., Corley J.C. (2012). Patch choice from a distance and use of habitat information during foraging by the parasitoid *Ibalia leucospoides*. Ecol. Entomol..

[19] Reeve J.D. (1987). Foraging behavior of *Aphytis melinus*: Effect of patch density and host size. Ecology.

[20] Weisser W.W., Houston A.I., Völkl W. (1994). Foraging strategies in solitary parasitoids: The trade-off between female and offspring mortality risks. Evol. Ecol..

[21] Cotes B., Rännbäck L.M., Björkman M., Norli H.R., Meyling N.V., Rämert B., Anderson P. (2015). Habitat selection of a parasitoid mediated by volatiles informing on host and intraguild predator densities. Oecologia.

[22] Milenovic M., Ripamonti M., Eickermann M., Rapisarda C., Junk J. (2023). Longevity of the whitefly parasitoid *Eretmocerus eremicus* under two different climate scenarios. Phytoparasitica.

[23] Van Driesche R.G., Lyon S.M., Hoddle M.S., Roy S., Sanderson J.P. (1999). Assessment of cost and performance of *Eretmocerus eremicus* (Hymenoptera: Aphelinidae) for whitefly (Homoptera: Aleyrodidae) control in commercial poinsettia crops. Fla. Entomol..

[24] Zilahi-Balogh G.M.G., Shipp J.L., Brodeur J. (2006). Influence of Light Intensity, Photoperiod, and Temperature on the Efficacy of Two Aphelinid Parasitoids of the Greenhouse Whitefly. Environ. Entomol..

[25] Zilahi-Balogh G.M.G., Shipp J.L., Cloutier C., Brodeur J. (2009). Comparison of searching behaviour of two aphelinid parasitoids of the greenhouse whitefly, *Trialeurodes vaporariorum* under summer vs. winter conditions in a temperate climate. J. Insect Behav..

[26] Polis G.A., Myers C.A., Holt R.D. (1989). The ecology and evolution of intraguild predation: Potential competitors that eat each other. Annu. Rev. Ecol. Syst..

[27] Yu H., Zhang Y., Kongming W., Gao W.X., Guo Y.Y. (2008). Field-Testing of Synthetic Herbivore-Induced Plant Volatiles as Attractants for Beneficial Insects. Environ. Entomol..

[28] Chailleux A., Mohl E.K., Alves M.T., Messelink G.J., Desneux N. (2013). Natural enemy-mediated indirect interactions among prey species: Potential for enhancing biocontrol services in agroecosystems. Pest Manag. Sci..

[29] Wajnberg E., Roitberg B.D., Boivin G. (2015). Using optimality models to improve the efficacy of parasitoids in biological control programmes. Entomol. Exp. Appl..

[30] Jonsson M., Kaartinen R., Straub C.S. (2017). Relationships between natural enemy diversity and biological control. Insect Sci..

[31] Culshaw-Maurer M., Sih A., Rosenheim J.A. (2020). Bugs scaring bugs: Enemy-risk effects in biological control. Ecol. Lett..

[32] Lange W.H., Bronson L. (1981). Insect pest of tomatoes. Annu. Rev. Entomol..

[33] Lloyd L. (1922). The control of the greenhouse whitefly (*Asterochiton vaporariorum*) with notes on its biology. Ann. Appl. Biol..

[34] Stansly P.A., Naranjo S.E., Brown J.K., Horowit A.R., Legg J.P., Polston J.E., Gerling D., Lapidot M. (2010). Bemisia: Bionomics and Management of a Global Pest.

[35] Capinera J. (2020). Handbook of Vegetable Pests.

[36] Rose M., Zolnerowich G. (1997). *Eretmocerus haldeman* (Hymenoptera: Aphelinidae) in the United States with Descriptions of New Species Attacking Bemisia (tabaci complex) (Homoptera: Aleyrodidae). Proc. Entomol. Soc..

[37] Ardeh M.J., Jong P.W., Van Lenteren J.C. (2005). Selection of Bemisia nymphal stages for oviposition or feeding, and host-handling times of arrhenotokous and thelytokous *Eretmocerus mundus* and arrhenotokous *E. eremicus*. BioControl.

[38] Brailovsky H. (2016). A review of the Geocoridae of Mexico (*Hemiptera*: *Heteroptera*: *Lygaeoidea*), with descriptions of four new species, new distributional records, and a key to the known subfamilies, tribes, genera, and species. Zootaxa.

[39] Leppla N.C., Johnson K.L. (2010). Guidelines for Purchasing and Using Commercial Natural Enemies and Biopesticides in Florida and Other States.

[40] Hunter C.D. (1997). Suppliers of Beneficial Organisms in North America.

[41] Cardona C., Rodríguez I., Bueno J., Tapia X. (2005). Biología y Manejo de la Mosca Blanca Trialeurodes vaporariorum en Habichuelas y Frijol.

[42] Headrick D.H., Bellows T.S., Perring T.M. (1996). Behaviors of Female *Eretmocerus* sp. nr. *californicus* (Hymenoptera: Aphelinidae) attacking *Bemisia argentifolii* (Homoptera: Aleyrodidae) on cotton, *Gossypium hirsutum*, (Malavaceae) and Melon, *Cucumis melo* (Cucurbitaceae). BioControl.

[43] Asplen M.K., Bellamy D.E., Byrne D.N. (2001). Eggs of *Eretmocerus eremicus*, a Whitefly Parasitoid. Vegetable Report.

[44] Cohen A.C. (1985). Simple Method for Rearing the Insect Predators *Geocoris punctipes* (Heteroptera: Lygaeidae) on a Meat Diet. J. Econ. Entomol..

[45] Champlain R.A., Sholdt L.L. (1967). Life history of *Geocoris punctipes* (Hemiptera: Lygaeidae) in the laboratory. Ann. Entomol. Soc. Am..

[46] Dumbar D.M. (1972). Notes on the mating behavior of *Geocoris punctipes* (Hemiptera: Lygaeidae). Ann. Entomol. Soc. Am..

[47] Miller J.R., Roelofs W.L. (1978). Sustained-flight tunnel for measuring insect responses to wind-borne sex pheromones. J. Chem. Ecol..

[48] Ardeh M.J. (2004). Whitefly Control Potential of *Eretmocerus eremicus* Prasitoids with Different Reproductive Modes. Ph.D. Thesis.

[49] Desneux N., Ramírez-Romero R., Bokonon-Ganta A.H., Bernal J.S. (2010). Attraction of the parasitoid *Cotesia marginiventris* to host (*Spodoptera frugiperda*) frass is affected by transgenic maize. Ecotoxicology.

[50] Guerrieri E. (2003). Flight behaviour of *Encarsia formosa* in response to plant and host stimuli. Entomol. Exp. Appl..

[51] Williams L., Rodriguez-Saona C., Castle S.C., Zhu S. (2008). EAG-Active Herbivore-Induced Plant Volatiles Modify Behavioral Responses and Host Attack by An Egg Parasitoid. J. Chem. Ecol..

[52] Ottoni E.B. (2000). EthoLog 2.2: A tool for the transcription and timing of behavior observation sessions. BRMIC.

[53] Wajnberg É., Bernhard P., Hamelin F., Boivin G. (2006). Optimal patch time allocation for time-limited foragers. Behav. Ecol. Sociobiol..

[54] Zar J. (2010). Biostatistical Analysis.

[55] Venables W.N., Ripley B.D. (2002). Modern Applied Statistics with S.

[56] R Core Team (2022). R: A Language and Environment for Statistical Computing; R Foundation for Statistical Computing.

[57] Silva D.B., Weldegergis B.T., Van Loon J.J.A., Bueno V.H.P. (2017). Qualitative and Quantitative Differences in Herbivore-Induced Plant Volatile Blends from Tomato Plants Infested by Either *Tuta absoluta* or *Bemisia tabaci*. J. Chem. Ecol..

[58] Loughrin J.H., Manukian A., Heath R.R., Turlings T.C.J., Tumlinson J.H. (1994). Diurnal cycle of emission of induced volatile terpenoids by herbivore-injured cotton plants. Plants Biol..

[59] Kugimiya S., Shimoda T., Tabata J., Takabayashi J. (2010). Present or past herbivory: A screening of volatiles released from *Brassica rapa* under caterpillar attacks as attractants for the solitary parasitoid, *Cotesia vestalis*. J. Chem. Ecol..

[60] Copolovici L., Kännaste A., Remmel T., Vislap V., Niinemets Ü. (2011). Volatile emissions from *Alnus glutinosa* induced by herbivory are quantitatively related to the extent of damage. J. Chem. Ecol..

[61] Miresmailli S., Gries R., Gries G., Zamar R.H., Isman M.B. (2011). Population density and feeding duration of cabbage looper larvae on tomato plants alter the levels of plant volatile emissions. Pest. Manag. Sci..

[62] McCormick A.C., Boeckler G.A., Köllner T.G., Gershenzon J., Unsicker S. (2014). The timing of herbivore-induced volatile emission in black poplar (*Populus nigra*) and the influence of herbivore age and identity affect the value of individual volatiles as cues for herbivore enemies. Plant Biol..

[63] Liu Y.Q., Thiel A., Hoffmeister T.S. (2009). Odor-mediated patch choice in the parasitoid *Venturia canescens*: Temporal decision dynamics. Entomol. Exp. Appl..

[64] Gohole L.S., Overholt W.A., Khan Z.R., Vet L.E.M. (2003). Role of volatiles emitted by host and non-host plants in the foraging behaviour of *Dentichasmias busseolae*, a pupal parasitoid of the spotted stemborer *Chilo partellus*. Entomol. Exp. Appl..

[65] Conchou L., Lucas P., Meslin C., Proffit M., Staudt M., Renou M. (2019). Insect Odorscapes: From Plant Volatiles to Natural Olfactory Scenes. Front. Physiol..

[66] Hassell M.P., Southwood T.R.E. (1978). Foraging strategies of insects. Annu. Rev. Ecol. Evol. Syst..

[67] Wäschke N., Meiners T., Rostás M., Wajnberg E., Colazza S. (2013). Foraging strategies of parasitoids in complex chemical environments. Chemical Ecology of Insect Parasitoids.

[68] Renou M., Anton S. (2020). Insect olfactory communication in a complex and changing world. Curr. Opin. Insect Sci..

[69] Chan H.K., Hersperger F., Marachlian E., Smith B.H., Locatelli F., Szyszka P., Thomas N.X. (2020). Odorant mixtures elicit less variable and faster responses than pure odorants. PLoS Comput. Biol..

[70] Riffell J.A., Shlizerman E., Sanders E., Abrell L., Medina B., Hinterwirth A.J., Kutz J.N. (2014). Sensory biology. Flower discrimination by pollinators in a dynamic chemical environment. Science.

