# Supplementary material for: Can Eretmocerus eremicus Assess Oviposition Sites with Varying Host Densities and Predation Risks, and Make Decisions Based on Scent Cues?
Source: Insects. 2026 Mar 17;17(3):329. doi: 10.3390/insects17030329 (PMC13027198; doi:10.3390/insects17030329)
Supplement: Supplementary file 1 [file insects-17-00329-s001.zip › insects-4094828-supplementary.pdf]

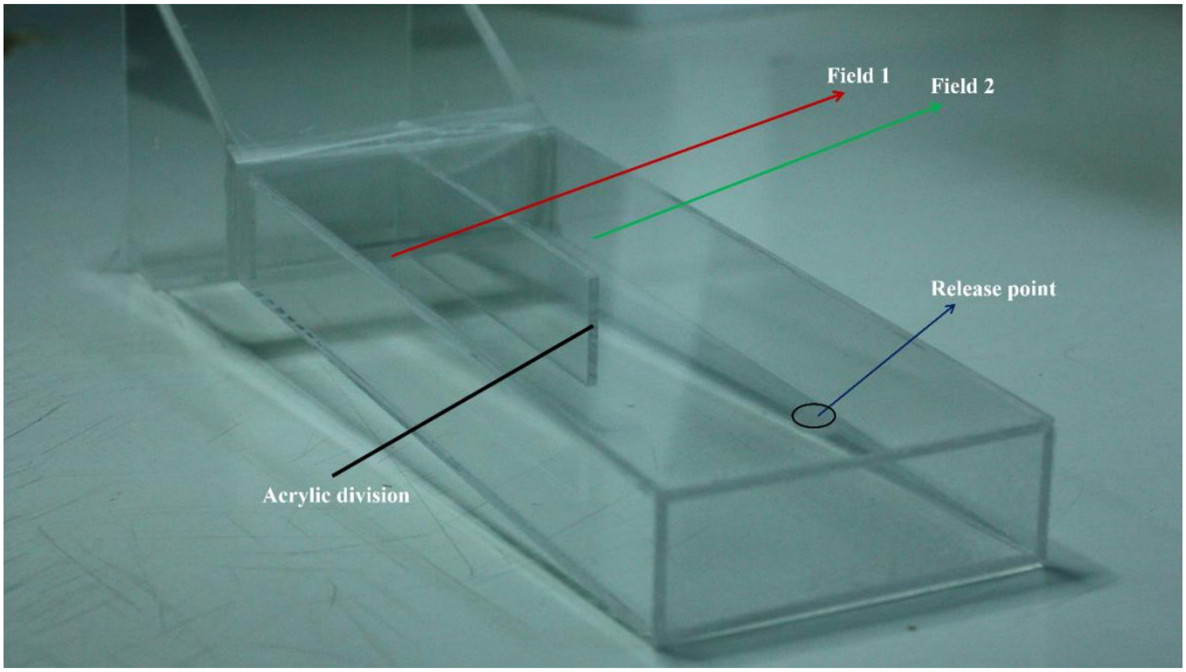

Figure S1A. Flight tunnel with sections depicted, including the release point (blue line), acrylic division (black line), field 1 (red line), and field 2 (green line). Photo: Dr. Chavarín-Gómez.

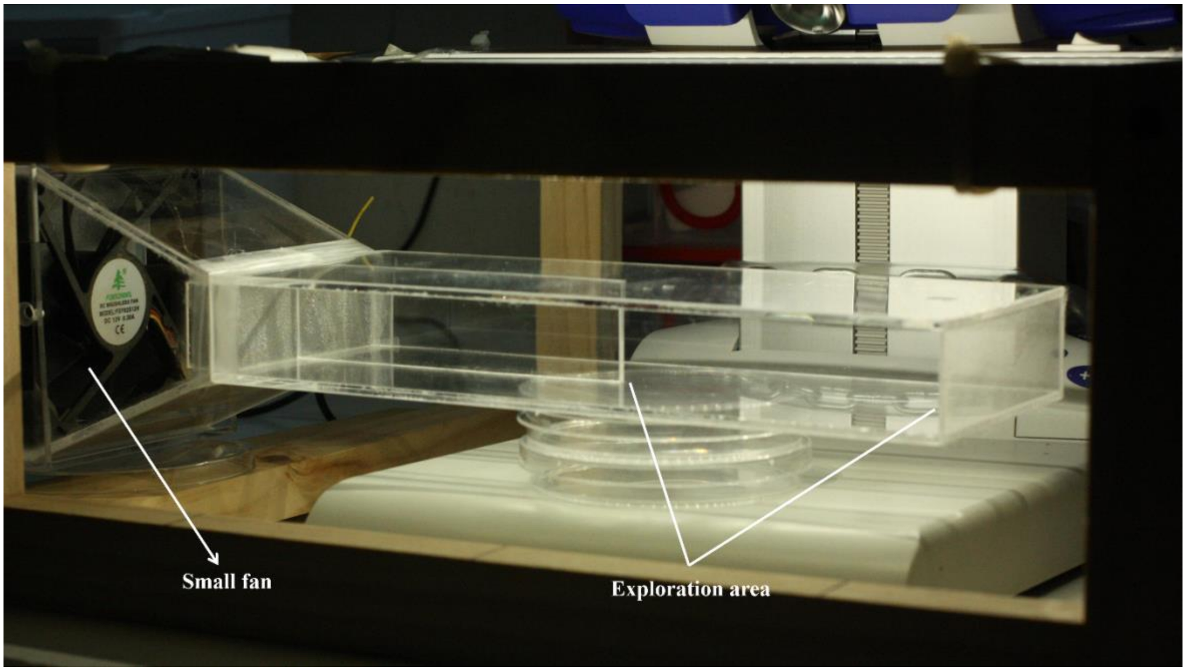

Figure S1B. Flight tunnel placed over the stereoscope with the lighting system. The fan location is visible, as is the area where the parasitoid can explore the scents associated with the offered patches. Photo: Dr. Chavarín-Gómez.
